# Non-Chloride in Situ Preparation of Nano-Cuprous Oxide and Its Effect on Heat Resistance and Combustion Properties of Calcium Alginate

**DOI:** 10.3390/polym11111760

**Published:** 2019-10-27

**Authors:** Peiyuan Shao, Peng Xu, Lei Zhang, Yun Xue, Xihui Zhao, Zichao Li, Qun Li

**Affiliations:** 1 College of Chemistry and Chemical Engineering, Qingdao University, Qingdao 266071, China; 15666681178@163.com (P.S.); X1627271005@163.com (P.X.); 2017020832@qdu.edu.cn (L.Z.); xueyun@qdu.edu.cn (Y.X.); zhaoxihui@qdu.edu.cn (X.Z.); 2 College of Life Sciences, Institute of Advanced Cross-Field Science, Qingdao University, Qingdao 266071, China; 3 Shandong Collaborative Innovation Center of Marine Biobased Fibers and Ecological Textiles, Qingdao University, Qingdao 266071, China

**Keywords:** alginate, non-chloride in situ preparation, nano-cuprous oxide, flame-retardant, mechanism

## Abstract

With Cu^2+^ complexes as precursors, nano-cuprous oxide was prepared on a sodium alginate template excluded of Cl^−^ and based on which the calcium alginate/nano-cuprous oxide hybrid materials were prepared by a Ca^2+^ crosslinking and freeze-drying process. The thermal degradation and combustion behavior of the materials were studied by related characterization techniques using pure calcium alginate as a comparison. The results show that the weight loss rate, heat release rate, peak heat release rate, total heat release rate and specific extinction area of the hybrid materials were remarkably lower than pure calcium alginate, and the flame-retardant performance was significantly improved. The experimental data indicates that nano-cuprous oxide formed a dense protective layer of copper oxide, calcium carbonate and carbon by lowering the initial degradation temperature of the polysaccharide chain during thermal degradation and catalytically dehydrating to char in the combustion process, and thereby can isolate combustible gases, increase carbon residual rates, and notably reduce heat release and smoke evacuation.

## 1. Introduction

Nano-cuprous oxide is a typical p-type semiconductor material with active electron holes, which exhibits effective catalytic activity, strong adsorption and low-temperature paramagnetic properties [1]. It has potential applications in organic synthesis, photoelectric conversion, new energy, water decomposition, sterilization, superconductivity, and others, and has become one of the most widely studied inorganic nanomaterials in the past decade [2,3,4,5,6,7,8,9,10].

**Figure 1:**
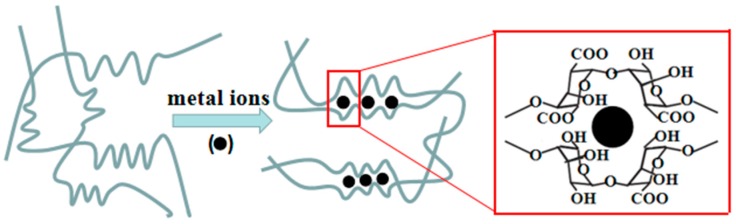
The crosslinking of sodium alginate—the “egg box” structure.

Due to their low cost and sustainability, marine biomaterials from renewable resources have attracted attention today as fossil energy is depleting. Alginate is a linear, binary-block copolymer with special structures and properties [11,12]. It has served as a template for the preparation of new functional hybrid materials in recent years [13,14,15,16,17]. In particular, sodium alginate can form a strong and stable “egg box” model (Figure 1) [12,18,19] structure of water-insoluble materials by crosslinking with divalent or trivalent metal ions, which is not only widely used in food and medicine [20,21,22], but also as an eco-friendly flame-retardant material [23,24,25,26,27].

It is well known that the goal of developing hybrid materials is to improve the functionality of the hybrid material by creating a synergistic effect through the interaction between two phases [13,28,29,30], and the flame-retardant properties of the inherently flame-retardant calcium alginate can be further improved by adding inorganic nanoparticles [31,32,33,34,35]. In order to overcome the defects caused by the agglomeration of inorganic nanoparticles due to the mechanical addition method [34], previous research on preparing calcium alginate-based hybrid materials by in situ methods have been reported [31,32,33]. Herein, a new multifunctional material employing nano-cuprous oxide to improve the antibacterial and catalytic properties of calcium alginate is presented. However, to the best of our knowledge, no such report has been well documented so far. Note that copper alginate shows no flame retardancy [36]; thus, the effect of nano-cuprous oxide on the flame-retardant properties of calcium alginate is worth exploring.

Generally, calcium chloride has been used as a crosslinking agent to prepare calcium alginate. However, since copper ions, cuprous ions, and chloride ions have complicated chemical reactions, it is difficult to prepare calcium alginate/nano-cuprous oxide hybrid materials by such a method based on the attempts in our laboratory for over a year. Therefore, we propose a novel “non-chlorine in situ” preparation method, aiming for the facile preparation of calcium alginate/nano-cuprous oxide hybrid materials by the in situ method in the absence of chloride ions. Our study found an improved limiting oxygen index of 54.0 for the hybrid material compared to pure calcium alginate. Although the role of inorganic nanoparticles in hybrid material has been reported so far, there has been no literature on the effects of nano-cuprous oxide on the flame-retardant properties of calcium alginate or its mechanism. The results here show that the peak heat release rate of calcium alginate can be reduced by 15% with only 1 wt % nano-cuprous oxide addition, and the flame-retardant mechanism was slightly different from the traditional mechanism [5,7]. In summary, the calcium alginate/nano-cuprous oxide hybrid materials prepared by the “non-chlorine in situ” method is a multi-functional material with important potential applications and flame-retardant, antibacterial, and catalytic properties. The effect and mechanism of cuprous oxide on the material is essential to the design of the material.

## 2. Materials and Methods

### 2.1. Materials

Sodium alginate (NaAlg) was purchased from the Guangfu Fine Chemical Research Institute (Tianjin, China). Pentahydrate copper sulfate (CuSO_4_·5H_2_O) was obtained from the Guangcheng Chemical Reagent Co., Ltd. (Tianjin, China). Both ammonia water (NH_3_·H_2_O) and sodium hydroxide (NaOH) were supplied by the fine chemical plant of the Laiyang Economic and Technological Development Zone (Yantai, China). Calcium acetate and ascorbic acid were purchased from Sinopharm Chemical Reagent Co., Ltd. (Shanghai, China). All the chemicals were of analytical grade and not further purified, and all the solutions were prepared using deionized water.

### 2.2. Preparation of Nano-Cu_2_O In Situ

**Scheme 1:**
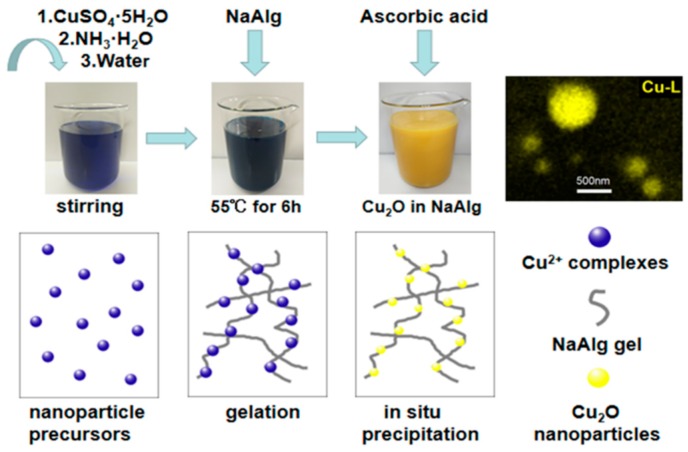
Schematic of nano-Cu_2_O preparation in situ.

The preparation process of the nano-Cu_2_O is shown schematically in Scheme 1. CuSO_4_·5H_2_O (3.9 g) was dissolved in a predetermined amount of deionized water, followed by the addition of 13 mL NH_3_·H_2_O (12 mol/L). Then, 20 g of NaAlg was added and the whole volume of the mixture solution was maintained at 500 mL. The solution was heated at a constant temperature of 55 °C for 6 h until a uniform and stable blue solution was formed. Subsequently, 11.4 g of ascorbic acid was added into the solution and was slowly stirred until the color entirely changed to yellow. Consequently, the nano-Cu_2_O was prepared on the NaAlg template.

### 2.3. Preparation of Calcium Alginate/Nano-Cuprous Oxide Hybrid Materials

NaOH (1 mol/L) was slowly dropped into the 3 wt % calcium acetate solution (2 L) until the pH reached 10. The as-prepared solution containing 4 wt % NaAlg was poured into molds of various shapes and then placed in the calcium acetate solution for 24 h, obtaining the crosslinked hybrid material (CaAlg/nano-Cu_2_O). Finally, the product was washed several times with deionized water and dried in a freeze-dryer for 24 h. It is found by calculation that the content of Cu_2_O in the hybrid material prepared by the in situ method was equivalent to the mechanical process of adding of 1 wt % Cu_2_O. As a comparison, pure calcium alginate (CaAlg) was prepared in a similar procedure only without the addition of the reagents related to the preparation of Cu_2_O.

### 2.4. Measurements and Characterizations

For the characterization of the calcined residue, the samples were heated to a target temperature (250, 450, and 750 °C) at a heating rate of 5 °C/min in air, and then kept for 2 h.

#### 2.4.1. Density

The quality and dimension of CaAlg and CaAlg/nano-Cu_2_O hybrid material were measured using an electronic balance (PL303 Precision Balance, Mettler-Toledo, Tokyo, Japan) and digital calipers, and then the densities were calculated based on the data.

#### 2.4.2. X-ray Diffraction (XRD)

An X-ray diffractometer (Ultima IV, Rigaku, Tokyo, Japan) was employed to investigate the samples and calcined residue under Cu Kα radiation (λ = 0.15418 nm), with a scan rate (2θ) of 5°/min in an angular range of 15–80 in continuous mode (operating voltage: 40 kV, current: 30 mA).

#### 2.4.3. Field Emission Scanning Electron Microscopy (FESEM)

The morphology and microstructure of the materials and their calcined residues were observed by a field emission scanning electron microscope (Sigma 500/VP, Carl Zeiss AG, Jena, Germany) at a beam voltage of 10 kV. Prior to SEM imaging, all the sample surfaces were sprayed with thin layers of gold to avoid charging under the electron beam. The distribution of Cu on the surface of the CaAlg/nano-Cu_2_O hybrid material was plotted using an energy dispersive X-ray spectrometer (EDX) attachment.

#### 2.4.4. Synchronous Thermal Analyzer (STA)

A synchronous thermal analyzer (TGA/DSC1 1600, Mettler Toledo, Columbus, OH, USA) was employed to analyze the materials under a nitrogen atmosphere. Samples of 4–6 mg were placed in an aluminum crucible and heated from room temperature to 1000 °C at a heating rate of 10 °C/min and a gas flow rate of 50 mL/min.

#### 2.4.5. Cone Calorimeter (CONE)

The combustion behavior of the samples was studied with a cone calorimeter (FTT-0007, Fire Testing Technology, East Grinstead, UK) under an external heat flux of 50 kW/m^2^ with horizontal orientation in air according to standard ISO 5660-1. The dimensions of all the samples were 100 mm × 100 mm × 8 mm.

#### 2.4.6. Limiting Oxygen Index (LOI)

The LOI values were determined using a M606B type instrument (Shanfang Instrument Co., Ltd., Qingdao, China). Samples of dimensions 80 mm × 10 mm × 8 mm were used for all the tests while CaAlg/nano-Cu_2_O was compressed to ensure that it was substantially the same quality as CaAlg. The samples were placed in a standard laboratory, at a temperature of 23 ± 2 °C and a relative humidity of 50 ± 5%, for at least 24 h before testing.

#### 2.4.7. Vertical Burning (UL-94)

Vertical burning tests were performed on a CZF-2 test instrument (Jiangning Analytical Instrument Factory, Nanjing, China) according to the standard American Society for Testing and Materials (ASTM) D3801. The size of both samples was 130 mm × 13 mm × 8 mm.

#### 2.4.8. Microscale Combustion Calorimeter (MCC)

A microscale combustion calorimeter (MCC-2, Govmark, Farmingdale, NY, USA) was used to investigate the flammability behavior of the test samples. Samples of 5 ± 0.05 mg were heated from room temperature to 750 °C (heating rate of 1 °C/s) in a stream of nitrogen flowing at 80 cm^3^/min. Before entering a 900 °C combustion furnace, the thermal degradation products were mixed with a 20 cm^3^/min steam, consisting of 20% oxygen and 80% nitrogen.

## 3. Results and Discussion

### 3.1. Morphology of Materials

**Figure 2:**
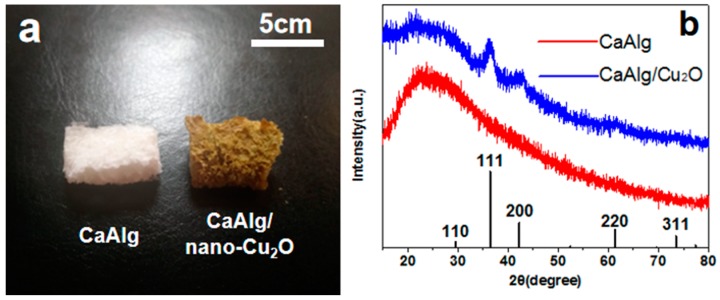
Digital photos (**a**) and X-ray diffraction (XRD) patterns (**b**) of CaAlg and CaAlg/nano-Cu_2_O.

In Figure 2a, the digital photo shows that the two materials exhibited similar porous structures with notably different colors. The difference in color can be attributed to the fact that nano-Cu_2_O changed the optical properties of the material itself, which was dependent on the structure, size, and dispersion of the particles [13]. The freeze-drying condensed the moisture in the material into ice, and the “ice matrix” left after the sublimation of the ice was the essence of its macroscopic appearance as a porous structure [37,38].

The two materials were analyzed by XRD and the results are shown in Figure 2b. Comparing the obtained diffraction peak with the pure phase Cu_2_O (cuprite, JCPDS-#99-0041), it was found that the four peaks with different diffraction values of two materials (at 36.4°, 42.3°, 61.4° and 73.5°) were identical with the crystal planes of Cu_2_O at (111), (200), (220), and (311), respectively. This confirms the existence of Cu_2_O and also the formation of hybrid materials.

**Figure 3:**
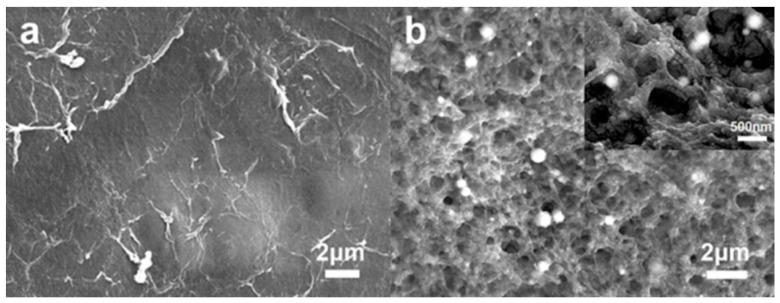
Scanning electron microscopy (SEM) images of (**a**) CaAlg and (**b**) CaAlg/nano-Cu_2_O. The inset in (**b**) is the magnified image.

It is noteworthy that the density of CaAlg was 0.19 g/cm^3^ while that of CaAlg/nano-Cu_2_O hybrid material was only 0.08 g/cm^3^. FESEM was used to study the morphology of the two materials in order to explain the difference in density. The results of electron microscopic characterization are shown in Figure 3. It can be seen from the analysis and comparison of the SEM images that CaAlg showed a lamellar structure with a smooth, uniform surface and a few nodules. CaAlg/nano-Cu_2_O was a three-dimensional network structure, and some spherical Cu_2_O particles with an average diameter below 100 nm were adhered to the CaAlg framework interface through a strong interaction, while the others were embedded in the framework of CaAlg.

It can be noticed from the microstructure that there were differing densities between the two materials and the three-dimensional network structure clearly exhibited a relatively lower density. The formation of this type of structure is due to the fact that NaAlg itself is rich in hydroxyl and carboxyl groups and can form intermolecular hydrogen bonds with Cu_2_O in situ [39,40]. The continuous network structure formed at the same time affected the mass transfer process of the solid–liquid phases in subsequent crosslinking, which led to the presence of a similar three-dimensional network structure in the final hybrid materials. This structure can effectively prevent the movement of polymer molecular chains during combustion with a lower thermal conductivity, and thus can provide better thermal stability of the materials [11,41].

### 3.2. Thermal Stability

**Figure 4:**
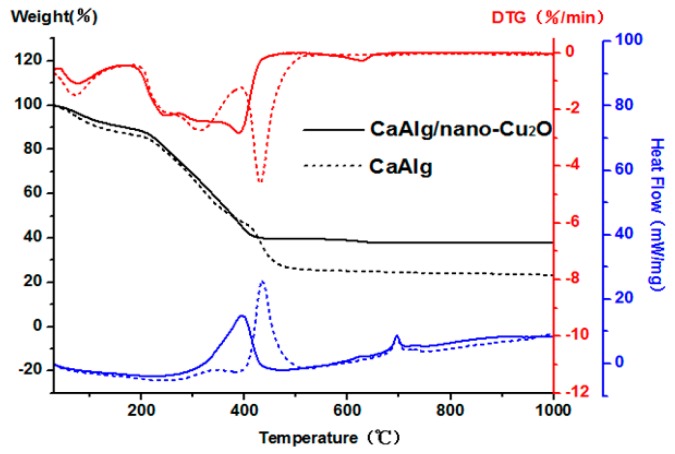
Weight loss, DTG, and DSC curves of CaAlg and CaAlg/nano-Cu_2_O under N_2_.

The thermal stability of CaAlg and CaAlg/nano-Cu_2_O was investigated by simultaneous thermal analysis (TG-DSC) and the comparison curves are displayed in Figure 4. The studied samples followed the same degradation trend and tended to be stable after three stages of decline. The decomposition process of CaAlg was similar to that of previous studies [31,42], and the three stages of its decomposition included: the desorption process of water at 50–140 °C, the thermal destruction of glycosidic bonds and the elimination process of adjacent hydroxyl groups at 240–400 °C; and a process took place in which the glycosidic bond was further destroyed to form an intermediate substance at 400–520 °C.

Comparing the two materials, the thermal stability of CaAlg/nano-Cu_2_O was obviously better than that of CaAlg, and the rate of weight loss was reduced by about 15%. In the desorption stage of water, the weight loss rate of CaAlg/nano-Cu_2_O was significantly lower than that of CaAlg, which can be attributed to the low thermal conductivity for the three-dimensional network structure of the hybrid material. As a biogenic polymer, CaAlg is rich in hydroxyl and carboxyl groups and can easily absorb water, while for hybrid materials, Cu_2_O can form multi-hydrogen bonds with the hydroxyl and carboxyl groups in CaAlg, so that the composites had a better thermal stability at low temperature.

As shown in Figure 4, more notable differences occurred at around 400 °C, in which CaAlg/nano-Cu_2_O was the first to re-decompose the glycosidic bonds at 360 °C, almost 40 °C less than CaAlg. This was mainly due to the fact that Cu_2_O in the hybrid material catalyzed the thermal decomposition of CaAlg and reduced the energy required for the reaction [43]. Comparing the DSC curves, it was found that although the decomposition reaction of CaAlg/nano-Cu_2_O proceeded in advance, the peak exotherm was significantly lower than that of CaAlg (namely 38.8%). The decrease in peak heat release indicated improvement in the thermal stability of the material.

In addition, there were two other noticeable observations in the curves shown in Figure 4. The slight weight loss of CaAlg/nano-Cu_2_O at 600 °C led to a weak change in the heat flux, which may be due to the decomposition of CaCO_3_ in the thermal cracking products of CaAlg catalyzed by CuO. It can be observed from the DSC curves that the two materials exhibited exothermic peaks at 700 °C without causing mass loss, which can be attributed to the phase transition process of the intermediate produced by the thermal decomposition of CaAlg at high temperature.

**Figure 5:**
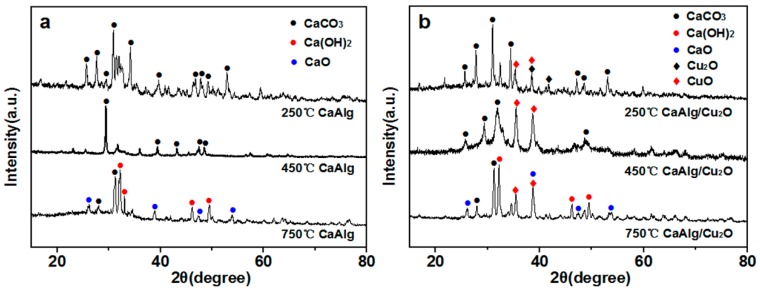
XRD patterns of calcined residue at different temperatures: (**a**) CaAlg and (**b**) CaAlg/nano-Cu_2_O hybrid material.

The XRD analysis of the calcined residue of the two prepared materials was carried out and the results are shown in Figure 5. It can be seen that CaAlg/nano-Cu_2_O showed a diffraction peak of Cu_2_O (cuprite, JCPDS-#99-0041) only in the calcination residue at 250 °C. However, in the calcination residue under the three temperature conditions, clear CuO (copper oxide, JCPDS-#89-5898) diffraction peaks can be observed, which were formed by the oxidation of Cu_2_O in the material. Except for Cu_2_O and CuO, the components of the calcined residue of the two materials were basically the same.

Among them, one category of product was CaCO_3_ of different crystal phases at 250 °C, including calcite (calcite, JCPDS-#99-0022), aragonite (aragonite, JCPDS-#71-2392) and vaterite (vaterite, JCPDS-#74-1867), while the product was at a single calcite phase of CaCO_3_ at 450 °C. With the decomposition of CaCO_3_ at 750 °C, not only CaCO_3_ but also CaO and Ca(OH)_2_ were generated.

**Figure 6:**
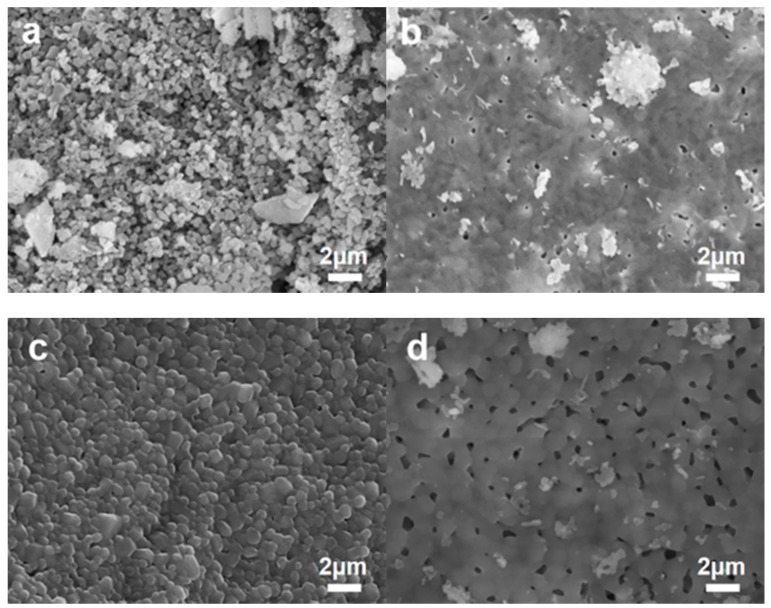
SEM images of calcined residue: (**a**) CaAlg heated to 250 °C; (**b**) CaAlg/nano-Cu_2_O heated to 250 °C; (**c**) CaAlg heated to 750 °C; (**d**) CaAlg/nano-Cu_2_O heated to 750 °C.

Through the morphological study of the residue after calcination of the two materials, the stability of the materials was analyzed from a microscopic point of view. The relevant characterization results are shown in Figure 6.

As shown in Figure 6a, CaCO_3_ particles of different sizes were distributed on the surface of the CaAlg calcination residue at 250 °C. However, for CaAlg/nano-Cu_2_O, the framework of CaAlg collapsed as the temperature rose and Cu_2_O particles with a higher surface energy were rapidly deposited on the surface of the material, which was oxidized to CuO. As seen in Figure 6b, Cu_2_O catalyzed the decomposition reaction of the outer layer of CaAlg, in which the release of gas carried off most of the heat and the remaining solid residue formed a dense barrier on the outer layer. The formation of the barrier layer can prevent heat and combustible gas from diffusing into the internal layer [44], greatly reducing the heat release rate and significantly improving the flame retardancy.

It can be observed from Figure 6c that dense polyhedral particles covered the surface of the CaAlg calcination residue at 750 °C, demonstrating the flame-retardant properties of the material. However, the most obvious morphological change in the calcination residue of CaAlg/nano-Cu_2_O was the inflation of the residue particles [45], as displayed in Figure 6d, which directly formed denser particle distribution and smaller surface voids. This can further improve the flame retardancy of the material. The morphological difference was related to the carbonized layer formation of the CuO-promoted material at high temperature [11,28].

### 3.3. Combustion Behavior

The experimental data of cone calorimetry are listed in Table 1. As seen, the heat release rate (HRR), peak heat release rate (PHRR), and total heat of release (THR) of CaAlg/nano-Cu_2_O were significantly lower than those of CaAlg. However, the quality of the samples used in this test was quite different due to the various structures, making the comparison of data inconclusive. At present, there is no known study on the influence of sample differences on cone calorimetry data and the degree of the relevant influence. Thus, the subsequent data were reanalyzed using an MCC experiment.

There are two quality-independent parameters in the CONE test—effective heat of combustion (EHC) and mean specific extinction area (mean SEA)—which are reliable results worthy of attention. EHC represents the ratio of HRR to mass loss rate, reflecting the degree of combustion of volatile gases in the gas phase flame. By comparison, it can be found that CaAlg/nano-Cu_2_O had a lower EHC value than that of CaAlg, indicating that the overall response of the former was lower than that of the latter, which indirectly proved that the hybrid material had better thermal stability. SEA is defined as the ability of a sample to decompose volatiles produced by flammable masses of combustibles. By comparing the mean SEA of the two materials, it can be clearly seen that CaAlg/nano-Cu_2_O exhibited better prominent smoke suppression ability than CaAlg.

Table 2 shows the results of the LOI and UL-94 tests for the two materials. In the UL-94 test, both materials were classified as V-0 based on the fact that both of them exhibited smoldering and maintained their original shape without melt dripping. For the limiting oxygen experiment, the materials are considered to be flame retardant when the LOI value surpasses 27.0. The LOI value of CaAlg was 49.2, which can be already recognized as well flame-retardant. Comparatively, the LOI value of CaAlg/nano-Cu_2_O reached 54.0, reflecting a successful improvement by introducing the nano-Cu2O particles to CaAlg.

MCC, also known as pyrolysis combustion flow calorimetry, is a standard ASTM test method (ASTM 7309) for the analysis of solid materials. It can dynamically measure the HRR and other relevant data based on the oxygen consumed during the experiment [24]. The material can be tested and analyzed at milligram sizes, which can effectively avoid the influence caused by differences in sample preparation [46].

As seen in Table 2, the MCC results are consistent with the phenomenon of the flame experiments. CaAlg had a HRC of 18 J/g/K, a PHRR of 16.19 W/g, and a THR of 4.3 kJ/g, all of which were at lower levels. The fabrication of CaAlg/nano-Cu_2_O further improved the flame retardancy of CaAlg, and the values of several parameters declined, especially for the PHRR, which was decreased by nearly 15%. At the same time, the 5% increase in residual carbon confirmed the effect of the nanoparticles on the catalytic carbon formation of CaAlg/nano-Cu_2_O as well. It is clear that nano-Cu_2_O showed positive influence on the flame retardancy of the hybrid materials.

### 3.4. Flame-Retardant Mechanism

**Scheme 2:**
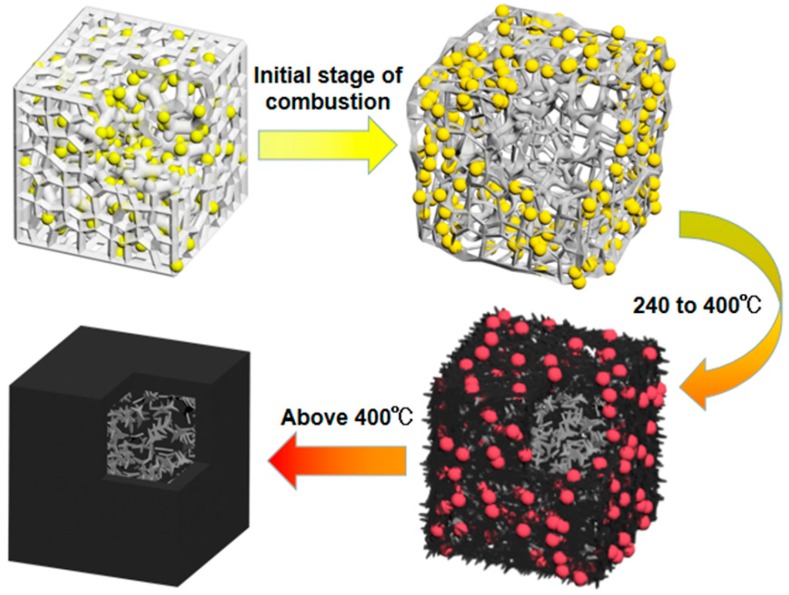
Flame-retardant mechanism of nano-Cu_2_O on the hybrid materials.

Scheme 2 shows the flame-retardant mechanism of nano-Cu_2_O on the CaAlg/nano-Cu_2_O hybrid material in detail. Due to the presence of inorganic nanoparticles, the hybrid materials formed a three-dimensional network structure with low thermal conductivity, which showed superior thermal stability at the initial stage of combustion.

At 40–400 °C, the skeleton of CaAlg gradually collapsed and disintegrated with the increase of temperature. The nano-Cu_2_O attached to the skeleton had a high surface energy and rapidly migrated and deposited on the surface of CaAlg/nano-Cu_2_O. Since Cu_2_O catalyzed the thermal decomposition reaction of CaAlg, the outermost CaAlg is the first to be decomposed at a relatively low temperature. Vapor and CO_2_ generated by dehydration and decarboxylation in the CaAlg thermal decomposition process carried away most of the heat, while the remaining solid residue, including CuO oxidized from Cu_2_O, formed a dense carbon barrier in the outermost layer of CaAlg/nano-Cu_2_O. The barrier layer prevented heat and flammable gas from diffusing into the inner layer, while the release of the incombustible gas lowered the concentration of the flammable gas at both the inner and outer contact surfaces, suppressing the overall thermal decomposition reaction to a lower degree and greatly reducing the HRR.

Above 400 °C, the thermal decomposition product CaCO_3_ began to shift to CaO. As the temperature increased further, char formation began. At this time, the CuO in the calcination residue of CaAlg/nano-Cu_2_O became a catalyst for the char formation reaction. A large amount of the carbonaceous carbon produced was coated on the surface of CaAlg/nano-Cu_2_O and appeared to be an expansion of the calcination residue. It promoted a denser barrier layer and a narrower surface void that both shielded the diffusion of flammable gases and blocked heat and heat radiation. The heat feedback of the material was reduced, and thereby the progress of the combustion reaction was suppressed. This further improved the flame retardancy of the hybrid materials.

## 4. Conclusions

In this study, nano-cuprous oxide was prepared by employing Cu^2+^ complexes as precursors via a non-chloride in situ method rather than the widely used direct crosslinking of the sodium alginate solution template. Then, the composites based on the calcium alginate/cuprous oxide nanoparticles were fabricated by a simple green crosslinking and freeze-drying process. Measurements and characterizations show that CaAlg/nano-Cu_2_O displayed a completely different three-dimensional network structure from CaAlg, with a LOI value up to 54.0. The addition of a mere 1 wt % nano-Cu_2_O reduced the PHHR by 15% and increased the residual carbon rate by 5%, which contributed to the improvement for the flame retardancy of CaAlg/nano-Cu_2_O. Moreover, it can be inferred from the supposed working and flame-retardant mechanism of nano-Cu_2_O on CaAlg that the carbon layer that formed at low temperature due to catalysis protected the internal material, reduced the HRR, THR, PHRR and EHC, and suppressed the release of smoke of the hybrid materials. The char formation was reinforced by CuO catalysis to reduce combustion performance and improve the flame-retardant properties of the material. CaAlg/nano-Cu_2_O not only has potential development prospects in the field of antibacterial and catalytic materials, but also a green material with lower heat release and smoke release, and improved flame-retardant properties compared to CaAlg.

## Figures and Tables

**Table 1:** CONE test results of CaAlg and CaAlg/nano-Cu_2_O. HRR = heat release rate; THR = total heat of release; PHRR = peak heat release rate; EHC = effective heat of combustion; SEA = specific extinction area.

Samples	TTI (s)	HRR (kW/m^2^)	THR (MJ/m^2^)	PHRR (kW/m^2^)	EHC (MJ/kg)	Mean SEA (m^2^/kg)
CaAlg	11	39.73	6.40	93.59	10.87	41.46
CaAlg/nano-Cu_2_O	6	16.09	2.02	67.30	9.14	21.43

**Table 2:** Limiting oxygen index (LOI) test, UL-94 test and MCC test results of CaAlg and CaAlg/nano-Cu_2_O.

Samples	LOI (%)	UL-94	HRC (J/g/K)	PHRR (W/g)	THR (kJ/g)	Residues (%)
CaAlg	49.2	V-0	18	16.19	4.3	55.65
CaAlg/nano-Cu_2_O	54.0	V-0	16	14.01	4	60.55
